# Broadening the Scope of Neural Network Potentials through Direct Inclusion of Additional Molecular Attributes

**DOI:** 10.1021/acs.jctc.4c01625

**Published:** 2025-02-11

**Authors:** Guillem Simeon, Antonio Mirarchi, Raul P. Pelaez, Raimondas Galvelis, Gianni De Fabritiis

**Affiliations:** † Computational Science Laboratory, 16770 Universitat Pompeu Fabra, Barcelona Biomedical Research Park (PRBB), C Dr. Aiguader 88, 08003 Barcelona, Spain; ‡ Acellera Labs, C Dr Trueta 183, 08005 Barcelona, Spain; § Institució Catalana de Recerca I Estudis Avançats (ICREA), Passeig Lluis Companys 23, 08010 Barcelona, Spain

## Abstract

Most state-of-the-art neural network potentials do not account for molecular attributes other than atomic numbers and positions, which limits its range of applicability by design. In this work, we demonstrate the importance of including additional electronic attributes in neural network potential representations with a minimal architectural change to TensorNet, a state-of-the-art equivariant model based on Cartesian rank-2 tensor representations. By performing experiments on both custom-made and public benchmarking data sets, we show that this modification resolves input degeneracy issues stemming from the use of atomic numbers and positions alone, while enhancing the model’s predictive accuracy across diverse chemical systems with different charge or spin states. This is accomplished without tailored strategies or the inclusion of physics-based energy terms, while maintaining efficiency and accuracy. These findings should furthermore encourage researchers to train and use models incorporating these additional representations.

## Graphical abstract

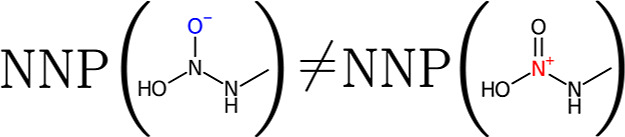

## 1 Introduction

Neural network potentials,^1–11^ which are deep learning models used to predict the energy and forces of atomic systems,^12^ have emerged as a tool making possible to circumvent the trade-off between computational cost and accuracy in computational chemistry. Despite its remarkable successes, these architectures typically rely on atomic numbers and positions as their only inputs, resulting in a simplification that overlooks significant quantum properties such as total charge and spin state by design. Neglecting these properties can lead to a degeneracy of inputs, where different molecular states, distinguishable only by their charge or spin, are represented identically by the neural network.

Given these considerations, the quest for extending the range of applicability of these models and for building general neural network potentials requires strategies making the incorporation of these additional attributes possible. For example, as a consequence of these current limitations, researchers have sometimes curated real-world data sets^13^ to avoid the inclusion of charged species,^14^ thereby restricting the diversity and realism of the training data, or directly avoided a special treatment of these attributes.^15^ To overcome these challenges, it is essential to integrate these properties.^16–18^

The incorporation of charges has been previously addressed in the literature to take into account the long-range effects that they induce in molecular systems.^5,18–24^ Previous approaches have typically involved mechanisms for the global redistribution of charge across the system, with neural networks informed by the chemical environment that predict intermediate properties such as electronegativities and effective charges, and equilibration schemes^25^ that solve systems of linear equations^19–22^ or use self-consistent processes.^26^ In other models^5,16^ additional quantities interpreted as atomic charges are predicted and used in ad-hoc introduced physics-based terms for energy prediction, such as Coulomb potential energies. Recently, the Latent Ewald Summation method^27,28^ proposed learning hidden charges from local features to compute long-range interactions, avoiding explicit charge equilibration while maintaining accuracy. While this approach can naturally handle long-range effects, an electrostatic-like interaction in reciprocal space is assumed.

In this work, we introduce a simple yet effective extension to a state-of-the-art model such as TensorNet,^29^ allowing it to accommodate charged molecules and spin states without requiring architectural changes or additional featurization, and therefore breaking the aforementioned degeneracy problem that most of the current models display. Despite not explicitly accounting for the global redistribution of properties, this enhancement retains the model’s original predictive accuracy without using additional physical terms or predicted quantities, being able to achieve state-of-the-art accuracy in common benchmark data sets. On top of this, since the presented method allows one to include these additional attributes in the learned representations, the prediction of other quantities used in the literature for the incorporation of long-range interactions is facilitated. Overall, the modification of TensorNet presented here expands its applicability to a broader range of chemical systems, addressing a critical shortcoming of most state-of-the-art architectures.

## 2 Method

Given some input atomic numbers and positions {*Z*, **r**}, TensorNet learns for every atom (*i*) a set of rank-2 tensors (3 × 3 matrices) *X* ^(*i*)^. TensorNet’s operations make these representations equivariant to rotations and reflections of the input, meaning that given some matrix *R* ∈ *O*(3), when the matrix is applied on input positions {**r**} → *R*{**r**}, atomic representations transform as *X* ^(*i*)^ → *RX* ^(*i*)^ *R* ^T^, where *R* ^T^ denotes the transpose of *R*. Furthermore, *X* ^(*i*)^ can be decomposed into scalar, vector, and symmetric traceless components, *I* ^(*i*)^, *A* ^(*i*)^ and *S* ^(*i*)^, respectively. After several message-passing layers, the squared Frobenius norms of the representations, which are invariant under *O*(3), are further processed by the neural network to predict energies, obtaining atomic forces via automatic differentiation.

One of the key operations in TensorNet, distinguishing it from other higher-rank spherical equivariant models, is how neighboring nodes’ tensor features are aggregated and used together with the ones of the receiving node to generate a new set of node tensor representations that transform appropriately under *O*(3). We refer the reader to ref 29 for full details and derivations. In each layer, after some node level transformations *X* ^(*i*)^ → *X*′^(*i*)^ = *X* ^(*i*)^/(∥*X* ^(*i*)^∥ + 1) → *Y* ^(*i*)^, pairwise messages *M* ^(*ij*)^ from neighbor (*j*) to receiving atom (*i*) are built by decomposing neighbor’s features *Y* ^(*j*)^

1 $$M^{\left(ij\right)}=\phi \left(r_{ij}\right)\left({f_{I}}^{ij}I^{\left(j\right)}+{f_{A}}^{ij}A^{\left(j\right)}+{f_{S}}^{ij}S^{\left(j\right)}\right)$$

where *f*_I_^*ij*^, *f*_A_^*ij*^, *f*_S_^*ij*^ are learnable functions of the distance between atoms *r*_*ij*_, and ϕ­(*r*_*ij*_) is a cosine cutoff function. Tensor messages are summed for all neighbors

2 $$M^{\left(i\right)}=\sum_{j\in N\left(i\right)}M^{\left(ij\right)}$$

and the generation of new features *Y*′^(*i*)^ from current node features *Y* ^(*i*)^ and corresponding message *M* ^(*i*)^ is performed via simple matrix product as

3 $$Y^{\prime \left(i\right)}=Y^{\left(i\right)}M^{\left(i\right)}+M^{\left(i\right)}Y^{\left(i\right)}$$

effectively mixing scalars, vectors, and tensors, and ensuring *O*(3)-equivariance, as proved in ref 29. Resulting representations are eventually manipulated to yield residual updates Δ*X*′^(*i*)^ to the layer’s input normalized features *X*′^(*i*)^

4 $$X^{\left(i\right)}\leftarrow X^{\prime \left(i\right)}+\Delta X^{\prime \left(i\right)}+{\left(\Delta X^{\prime \left(i\right)}\right)}^{2}$$

using *X* ^(*i*)^ to feed the following layer and restart the process.

We propose to include molecular states’ information ψ_*k*_, by modifying eqs 2 and 3 in TensorNet’s interaction layers in the following node-wise manner

5 $$Y^{\prime \left(i\right)}=\left(1+\lambda_{k}\psi_{k}\right)\left(Y^{\left(i\right)}M^{\left(i\right)}+M^{\left(i\right)}Y^{\left(i\right)}\right)$$

6 $$X^{\left(i\right)}\leftarrow X^{\prime \left(i\right)}+\Delta X^{\prime \left(i\right)}+\left(1+{\mathrm{\lambda ̃}}_{k}\psi_{k}\right){\left(\Delta X^{\prime \left(i\right)}\right)}^{2}$$

where $\lambda_{k},{\mathrm{\lambda ̃}}_{k}$ can be regarded as per-layer constant or learnable weights for the encoding of states such as total charge or spin ψ_k_ = {*Q*, *S*, ...}. Notice that the modification, while forcing the network to output different values when considering the same input atomic numbers and positions, does not break equivariance under *O*(3), since it amounts to a rescaling of tensor features by means of a state-dependent scalar factor. Furthermore, it has been designed in such a way that when the system is neutral and in its singlet state, i.e.

7 Q=S=0

the model defaults to the original TensorNet (eqs 2 and 3), therefore recovering the predictive accuracy already demonstrated in ref 29.

Note that since operations are performed node-wise, this framework naturally accommodates both atomic and molecular attributes. For atomic attributes (such as partial charges ψ_k_ = *q* ^(*i*)^), each node’s operations can be modified by its specific value. For molecular attributes (like total charge ψ_k_ = *Q* or spin ψ_k_ = *S*), the same value modifies operations across all nodes in the system. This allows incorporating either local or global information within the same mathematical framework. In the case of atomic charges, commonly used in biomolecular simulations, one needs to rely on some external partial charge computation scheme, which might assume the availability of information beyond atomic numbers and positions, such as SMILES representations, molecular bonds or other preprocessing steps. Furthermore, given the possibility of including total charge as an input to the network, our method also enables the prediction of additional atomic quantities that can be trained to match DFT partial charges and used to evaluate electrostatic terms, as previously done in the literature.^16,21,22^ Nevertheless, we will not make use of this capability in our experiments, showing that highly accurate models such as TensorNet do not require the inclusion of physics-based terms, such as Coulomb, dispersion or nuclear repulsion, for the benchmark systems studied in this work and commonly used in the literature.

Note that while this framework theoretically supports multiple attributes, careful consideration would be needed to handle them simultaneously. For example, one could consider the modification of eqs 5 and 6, and promote λ_k_ψ_k_ to a linear combination of attributes *∑*_k_λ_k_ψ_k_. However, fixed weights could lead to degeneracies, where distinct quantum states produce identical scalar factors. A more sophisticated approach using learnable weighting parameters λ_k_ could help discover encodings that properly distinguish different quantum states while preserving their physical influence. The current implementation demonstrates the effectiveness of individual attributes separately, leaving the exploration of simultaneous quantum attributes as future work.

## 3 Results and Discussion

We performed a series of experiments with the extended TensorNet model using the TorchMD-Net framework.^30^ We trained the model on a custom-made data set and several publicly available data sets, targeting atomic number and geometry degeneracy, as well as the simultaneous presence of different charge states regardless of structural or geometric overlap between these. We used a generic set of reasonable hyperparameters, without addressing data set-dependent fine-tuning to obtain the best possible performances. In all cases, we used the direct model prediction of energies and forces (when needed), without the inclusion of physics-based terms. Overall, we evaluate TensorNet’s extension when dealing with total charges, Gasteiger partial charges, or singlet and triplet states. Training details and hyperparameters used in the experiments can be found in the Supporting Information

### 3.1 Toy Degeneracy Problem

As a first test, to illustrate the input degeneracy issue and how TensorNet’s extension resolves it without affecting its baseline accuracy, we constructed two toy data sets, Data set A and Data set B, each one comprising five members of five pairs of unique molecular systems, each of the elements in the pair differing in total charge (see Figure 1). These pairs are indistinguishable from a neural network that does not account for total charge, as they share identical atomic numbers and geometric configurations. The data sets include total charges, Gasteiger partial charges computed with RDKit, and calculated energies and atomic forces for 2000 conformers per molecule using GFN2-xTB.^31^ Therefore, each data set contains a total of 10 k data points. Conformers were generated by minimizing each molecule, displacing atomic positions with Gaussian noise with a standard deviation of 0.2 Å, and filtering them such that maximum atomic forces are <100 eV/Å.

**Figure 1:**
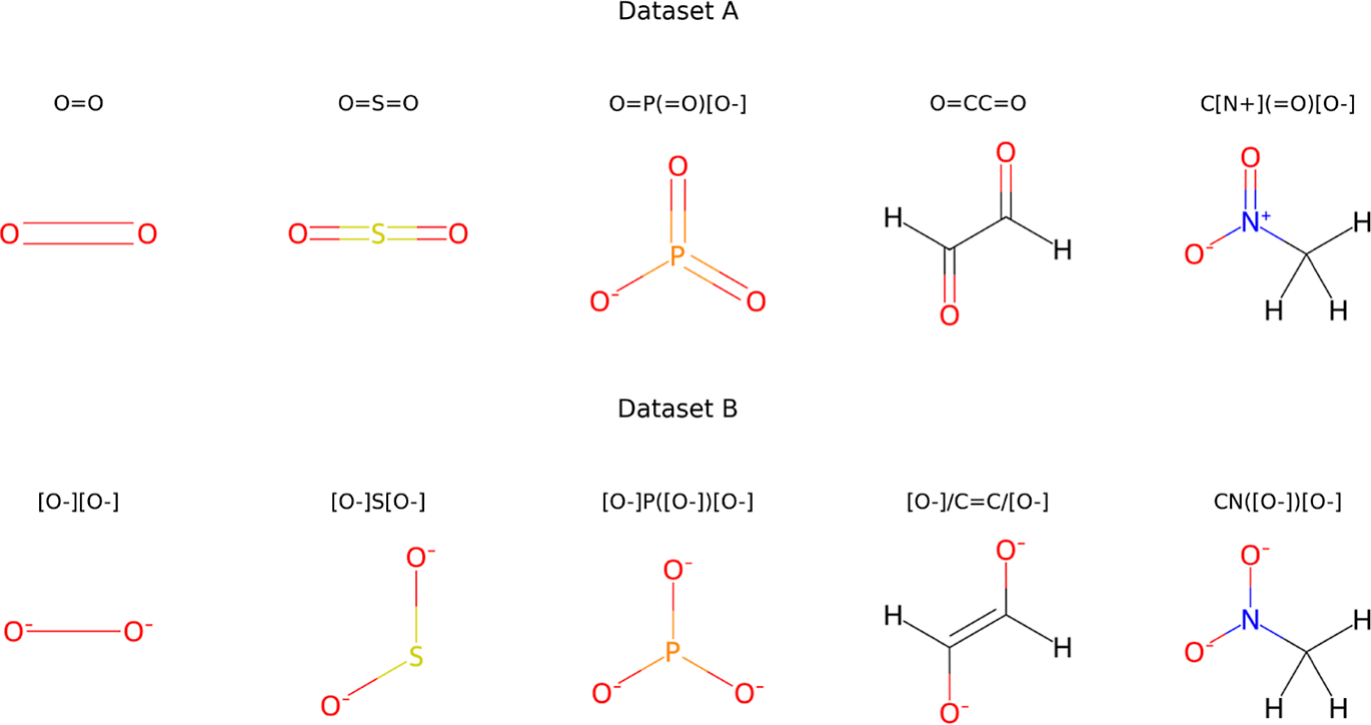
Molecules included in the A and B toy data sets, from which 2000 data points per molecule are obtained by generating conformers and computing potential energies and atomic forces using GFN2-xTB.^31^ Columns illustrate degenerate pairs of molecules for a neural network that uses solely atomic numbers and positions as inputs.

The logic behind the experiment is the following: when training the original TensorNet on each data set separately, the learning should proceed as expected, with the network successfully learning the mapping between the atomic inputs and the output properties. However, when the data sets are combined, the inability to distinguish charge states leads to an overlap in the input space, being mapped to different values of energies and forces. As a result, the network should fail to learn accurately. On the other hand, the extension of the model should allow the network to learn accurately the mapping between inputs and outputs in all cases.

Splits of 50/10/40% were used for training, validation, and testing, respectively, both for single and combined data sets. For the charge-aware case, we tried separately the inclusion of total charges *Q* and Gasteiger partial charges *q* ^(*i*)^, both with $\lambda_{Q}={\mathrm{\lambda ̃}}_{Q}$ and $\lambda_{q^{\left(i\right)}}={\mathrm{\lambda ̃}}_{q^{\left(i\right)}}$ equal to 0.1 across all layers. Results can be found in Table 1. As expected, the original model trained on the merged data set has a very poor accuracy (TensorNet, Data set A ∪ B). The extension with total and partial charges allows us to learn the merged data set with sub-meV and sub-meV/Å differences in energy and force errors with respect to single data set training of the corresponding extended model. The use of total charge *Q* gives better results than the use of partial charges *q* ^(*i*)^.

**Table 1:** Toy Datasets Results^a^

model		data set A	data set B	data set A ∪ B
TensorNet^29^	*E* (meV)	2.6	2.3	4437.0
	*F* (meV/Å)	11.6	14.3	772.0
TensorNet + 0.1*Q*	*E* (meV)	2.4	2.3	2.5
	*F* (meV/Å)	11.9	14.8	13.9
TensorNet + 0.1*q* ^(*i*)^	*E* (meV)	2.8	2.3	3.2
	*F* (meV/Å)	13.7	14.9	14.8

a Energy (*E*) and forces (*F*) mean absolute errors in meV and meV/Å. *Q* refers to total charge while *q* ^(*i*)^ refers to Gasteiger partial charges, with factors corresponding to weights $\lambda_{Q}={\mathrm{\lambda ̃}}_{Q}$ and $\lambda_{q^{\left(i\right)}}={\mathrm{\lambda ̃}}_{q^{\left(i\right)}}$ , respectively.

### 3.2 Spice PubChem

The PubChem subset inside the SPICE data set^13^ (version 1.1.4) consists of conformers of small drug-like molecules, with energies and forces computed at the ωB97M-D3­(BJ)/def2-TZVPPD level of theory. Approximately 4% of the molecules and conformers in the data set are not neutral. The data set already provides total charges, while we computed Gasteiger partial charges for all the molecules in the subset, including the neutral ones. The computation of partial charges failed with RDKit for 42 molecules, with corresponding conformers being discarded. This resulted in 707,558 data points in total. To include total charge *Q* or atomic partial charges *q* ^(*i*)^, we take $\lambda_{Q}={\mathrm{\lambda ̃}}_{Q}$ and $\lambda_{q^{\left(i\right)}}={\mathrm{\lambda ̃}}_{q^{\left(i\right)}}$ constant and nonlearnable across all layers, equal to 0.1 or 0.25. We used 80/10/10% splits for training, validation, and testing, respectively.

In Table 2, we show that including total charge *Q* or partial charges *q* ^(*i*)^ can improve energy and forces accuracy. Even though charged species comprise only 4% of the data points, the freedom given to the model to adapt to varying charge states improves significantly the accuracy on both charged and neutral conformations. With the best method, which uses total charge *Q* with $\lambda_{Q}={\mathrm{\lambda ̃}}_{Q}=0.1$ , reductions of 30% and 12% in energy errors are achieved for charged and neutral test molecules, respectively. The persisting performance gap between neutral and charged molecules (Table 2) can be attributed to data set imbalance. This is evidenced by our toy data set experiments (Table 1), where an equal representation of charge states (2000 conformations each) shows no accuracy degradation for the merged data set. In fact, force prediction accuracy for the merged data set falls between that of individual data sets, indicating comparable performance across charge states when properly balanced. The imbalanced case can be understood as model parameters being predominantly trained on *Q* = 0 data, with a limited representation of other charge values. Nevertheless, even with just 4% charged species, including total charge consistently improves overall and charge-specific accuracy. This consistent improvement in prediction accuracy for both neutral and charged species suggests that the development of distinct internal representations due to charge awareness benefits the model beyond just handling charged molecules. This demonstrates that incorporating attributes like total charge helps the model develop more physically meaningful representations, enhancing predictive performance across all molecular states. The inclusion of Gasteiger partial charges gives relevant out-of-the-box improvements too.

**Table 2:** SPICE PubChem Results^a^

model		test total	test neutral	test charged
TensorNet^29^	*E* (meV)	34.2	32.7	70.1
	*F* (meV/Å)	34.7	33.8	58.4
TensorNet + 0.1*q* ^(*i*)^	*E* (meV)	31.5	30.5	55.2
	*F* (meV/Å)	33.9	33.0	56.7
TensorNet + 0.25*q* ^(*i*)^	*E* (meV)	30.5	29.6	50.4
	*F* (meV/Å)	32.9	32.1	55.6
TensorNet + 0.1*Q*	*E* (meV)	**29.4**	**28.6**	**49.3**
	*F* (meV/Å)	**31.4**	**30.6**	**50.1**
TensorNet + 0.25*Q*	*E* (meV)	29.6	**28.6**	52.0
	*F* (meV/Å)	31.6	**30.6**	52.9

a Energy (*E*) and forces (*F*) mean absolute errors in meV and meV/Å, on the entire test set, and separately on neutral and charged conformations in the test set. *Q* refers to total charge while *q* ^(*i*)^ refers to Gasteiger partial charges, with factors corresponding to weights $\lambda_{Q}={\mathrm{\lambda ̃}}_{Q}$ and $\lambda_{q^{\left(i\right)}}={\mathrm{\lambda ̃}}_{q^{\left(i\right)}}$ , respectively.

### 3.3 Carbon Chain, Silver Clusters and Sodium Chloride Clusters

These data sets were introduced in ref 21 to illustrate the shortcomings of previous generations of models,^32,33^ and are typically used as benchmarks for architectures incorporating long-range interactions and electrostatics, allowing us to compare their performances against the modified TensorNet, which does not incorporate physics-based terms in the energy prediction.

#### 3.3.1 Carbon Chain, **C**_10_ **H**_2_/**C**_10_ **H**_3_^+^

This benchmark consists of two subsystems: a neutral linear carbon chain with hydrogen atoms at each end, C_10_H_2_, and a protonated counterpart, C_10_H_3_^+^, yielding a total charge of +1 and resulting in a global charge redistribution.

#### 3.3.2 Silver Clusters, **Ag**_3_^±^

In this case, the data set consists of triangular and linear Ag trimers with total charges of +1 and −1, respectively. These systems define an ill-posed learning problem for models which are unaware of charge in a similar way to the toy data sets from Section 3.1.

#### 3.3.3 Sodium Chloride Clusters, **Na**_8/9_ **Cl**_8_^+^

This benchmark contains a positively charged Na_9_Cl_8_^+^ cluster and the system resulting from the removal of a neutral sodium atom, Na_8_Cl_8_^+^. The +1 total charge of the cluster is maintained, resulting in a redistribution of charge when the sodium atom is removed.

In line with previous work, we trained extended TensorNet on 90% data points, being the remaining ones used as a separate validation set. Comparative results for 4D-HDNNP,^21^ PANNA-LR,^22^ CACE-LR,^28^ SpookyNet^16^ and TensorNet can be found in Table 3. In the case of 4G-HDNNP, PANNA-LR (γ_q_ > 0) and SpookyNet, the models are trained to reproduce DFT atomic reference charges and include an explicit electrostatic term in the energy prediction that makes use of these. In SpookyNet, nuclear repulsion and D4 dispersion terms are also included. We emphasize that, in contrast, TensorNet was directly trained on energy and force labels without making use of any charge predictions or prior physical terms. CACE-LR makes use of Latent Ewald Summation,^27^ which learns hidden “latent charges” from atomic features without explicit reference to any specific charge definition, and uses these to compute long-range interactions via Ewald summation while avoiding the need for charge equilibration. However, one can consider the assumed interaction in reciprocal space as an electrostatic-like ansatz. Therefore, we compared our approach to a wide diversity of methods in the literature.

**Table 3:** Charged Benchmark Systems C_10_H_2_/C_10_H_3_^+^, Ag_3_^±^ and Na_8/9_Cl_8_^+^ ^a^

model		C_10_H_2_/C_10_H_3_^+^	Ag_3_^±^	Na_8/9_Cl_8_^+^
4G-HDNNP^21^	*E* (meV/atom)	1.194	1.323	0.481
	*F* (meV/Å)	78.00	31.69	32.78
PANNA-LR^22^	*E* (meV/atom)	1.17	0.80	0.40
	*F* (meV/Å)	79	**20**	19
CACE-LR^28^	*E* (meV/atom)	0.73	**0.162**	0.21
	*F* (meV/Å)	36.9	29.0	9.78
SpookyNet^16^	*E* (meV/atom)	0.364	0.220	0.135
	*F* (meV/Å)	5.802	26.64	**1.052**
TensorNet	*E* (meV/atom)	**0.124** (0.001)	1683.11 (203.36)	**0.103** (0.003)
	*F* (meV/Å)	**1.88** (0.02)	912.67 (39.20)	1.73 (0.06)
TensorNet + 0.1*Q*	*E* (meV/atom)	**0.124** (0.001)	0.888 (0.019)	**0.102** (0.003)
	*F* (meV/Å)	**1.90** (0.15)	**20.70** (0.77)	1.78 (0.09)

a Energy (*E*) and forces (*F*) root mean squared errors in meV/atom and meV/Å, with standard deviation over 3 splits between parentheses for TensorNet, on the validation set consisting of 10% of data points. *Q* refers to total charge, with factor corresponding to weights $\lambda_{Q}={\mathrm{\lambda ̃}}_{Q}=0.1$ . Results for 4G-HDNNP and SpookyNet have been taken from 16, PANNA-LR results have been extracted from 22 corresponding to the best-performing model trained on partial charges, γ_q_ > 0 as denoted in the reference, while CACE-LR results have been obtained from ref 28.

Our model performs competitively or outperforms SpookyNet, the current best performing architecture. The baseline TensorNet already shows strong performance on the C_10_H_2_/C_10_H_3_^+^ and Na_8/9_Cl_8_^+^ systems even without charge awareness. This is because these systems can be distinguished structurally: C_10_H_3_^+^ has an additional proton compared to C_10_H_2_, while Na_9_Cl_8_^+^ has one more sodium atom than Na_8_Cl_8_^+^. However, for Ag_3_^±^, where three silver atoms form either a cation or anion, the baseline model fails to distinguish between charge states, leading to large errors (1683.11 meV/atom). The inclusion of charge awareness maintains the strong performance on structurally distinguishable systems while dramatically improving predictions for Ag_3_^±^ (0.888 meV/atom), demonstrating its ability to resolve input degeneracy when geometric information alone is insufficient.

Furthermore, we compared the speed performance of SpookyNet and TensorNet for a carbon chain conformation, using the ASE Calculator implementation^34,35^ for SpookyNet and the TorchMD-Net external calculator for TensorNet. The speed benchmark was conducted on an NVIDIA RTX 4090, with both models calculating energy and forces over 100 runs. TensorNet achieved a mean runtime of 1.462 ms per inference step, while SpookyNet recorded 1.751 ms. Notably, while SpookyNet’s long-range electrostatics scale as *O*(*N* ^2^), our method scales as *O*(*N*).

### 3.4 Solvated Protein Fragments

The solvated protein fragments data set, introduced in ref 5, includes structures for all possible hydrogen-saturated covalently bonded fragments with up to eight heavy atoms (C, N, O, S) derived from proteins. It accounts for different charge states of amino acids due to (de)­protonation, with total charges up to ±2. The data set features solvated variants with varying water molecule numbers, and randomly sampled dimer interactions of protein fragments, along with pure water structures with up to 40 molecules. Multiple conformations are included, resulting in more than 2.7 M structures with reference energies, forces, and dipole moments.

In line with ref 5, we trained TensorNet with the inclusion of total charge $\left(\lambda_{Q}={\mathrm{\lambda ̃}}_{Q}=0.1\right)$ on the energy and forces, excluding dipoles, of 2560 k structures, with 100 k of these being used for validation, being the remaining ones used for testing. Results are found in Table 4, and demonstrate that the modified TensorNet significantly outperforms the more accurate ensemble of five PhysNet models, which use explicit electrostatic and dispersion energy terms in the predictions, particularly in energies, nearly halving its error.

**Table 4:** Solvated Protein Fragments Results^a^

	PhysNet	PhysNet-ens5	TensorNet + 0.1*Q*
*E*	1.03 kcal/mol	0.95 kcal/mol	**0.55** (0.04) kcal/mol
*F*	0.88 kcal/mol/Å	0.72 kcal/mol/Å	**0.63** (0.09) kcal/mol/Å

a Comparison of mean absolute errors on the solvated protein fragments test set for energies (*E*) and forces (*F*). *Q* refers to total charge, with the factor corresponding to $\lambda_{Q}={\mathrm{\lambda ̃}}_{Q}=0.1$ . PhysNet-ens5 denotes an ensemble of five PhysNet models. Results have been taken from ref 5.

### 3.5 QMspin

The QMspin data set, introduced in ref 36, includes molecules optimized in both singlet and triplet states. The data set, drawn from the QM9 database, provides a comprehensive collection of around 13,000 carbene structures, with energies calculated for both singlet and triplet states. Therefore, QMspin contains approximately 26,000 data points. The singlet and triplet energy information enables the assessment of the model’s predictive performance on spin state differences, an aspect previously unaddressed due to the degeneracy of inputs, allowing us to test and validate TensorNet’s extension beyond charged states. We incorporate singlet or triplet states as *S* = 0 and *S* = 1, respectively, and $\lambda_{S}={\mathrm{\lambda ̃}}_{S}=0.1$ nonlearnable for all layers.

We removed 228 data points, which corresponded to geometry files where the number of atoms in the header did not match the number of atoms with coordinate entries. Following ref 16, we used 20 k and 1 k data points for training and validation, respectively, the remaining data points being used for testing. The results, found in Table 5, show that the inclusion of the spin state *S*, and therefore degeneracy breaking, improves 10-fold the accuracy of the model, achieving 43 meV error in energies, considered chemical accuracy. Furthermore, this represents an improvement of ∼40% with respect to SpookyNet,^16^ the only model that to the best of our knowledge has been benchmarked against the QMspin data set. Again, SpookyNet models explicitly electrostatic, dispersion, and nuclear repulsion interactions.

**Table 5:** QMspin Results^a^

	SpookyNet^16^	TensorNet	TensorNet + 0.1*S*
*E*	68 meV	432 (19) meV	**43** (2) meV

a Comparison of mean absolute errors on the QMspin test set energies (*E*), in meV. *S* refers to spin state, with the factor corresponding to $\lambda_{S}={\mathrm{\lambda ̃}}_{S}=0.1$ .

## 4 Conclusion

In this work, we have demonstrated and addressed a significant gap in some state-of-the-art neural network potentials, which typically disregard charge and spin states in the model’s representations. We modify TensorNet to accommodate these features with a zero-cost architectural change which effectively resolves degeneracy issues arising from the omission of these critical quantum attributes. Moreover, through experiments on well-established and custom-made benchmark data sets, we have shown that the predictive accuracy of the model is improved and that it performs on par with or outperforms existing methods that require more complex treatments of charges and spins.

Our results highlight that even a straightforward incorporation of these properties can lead to substantial improvements without the need for the introduction of additional physical terms or predicted quantities, despite facilitating and not precluding their use. Future work could explore more sophisticated strategies for scaling additional attributes, such as making them learnable scalars dependent on atomic properties.

Although this work focuses mainly on global attributes like total charge and spin, our framework mathematically supports atomic-level attributes like partial charges that could better represent local chemical environments in more complex and heterogeneous systems. However, this capability would require thorough validation and benchmarking beyond the scope of this work. Similarly, though our model effectively handles charge effects through implicit learning, it does not guarantee the correct asymptotic behavior of electrostatic interactions that physical laws demand, trading exact long-range physics for architectural simplicity while maintaining strong performance within typical molecular scales.

Overall, given the strong performance exhibited in our results, this work emphasizes the importance of incorporating charge and spin states to achieve high predictive accuracy and generality in modeling chemical systems.

## Supplementary Material



## Data Availability

TensorNet and its extension can be found within TorchMD-Net: https://github.com/torchmd/torchmd-net. The toy data sets have been made available at https://zenodo.org/records/10852523. The SPICE data set version 1.1.4 is publicly available at https://zenodo.org/records/8222043. The QMspin data set is available at https://archive.materialscloud.org/record/2020.0051/v1.

## Notes

Training and hyperparameters used for the different experiments presented throughout the manuscript is available (PDF)
