# Supplementary material for: Broadening the Scope of Neural Network Potentials through Direct Inclusion of Additional Molecular Attributes
Source: J Chem Theory Comput. 2025 Feb 11;21(4):1831–7. doi: 10.1021/acs.jctc.4c01625 (PMC12131224; doi:10.1021/acs.jctc.4c01625)
Supplement: Supplementary file 1 [file ct4c01625_si_001.pdf]

Supplementary Information: Broadening the Scope of Neural Network Potentials through Direct Inclusion of Additional Molecular Attributes

Guillem Simeon,† Antonio Mirarchi,† Raul P. Pelaez,† Raimondas Galvelis,†,‡ and Gianni De Fabritiis*,†,‡,¶

†*Computational Science Laboratory, Universitat Pompeu Fabra, Barcelona Biomedical Research Park (PRBB), C Dr. Aiguader 88, 08003 Barcelona, Spain.*
‡*Acellera Labs, C Dr Trueta 183, 08005, Barcelona, Spain*
¶*Institució Catalana de Recerca i Estudis Avançats (ICREA), Passeig Lluis Companys 23, 08010 Barcelona, Spain*

E-mail: g.defabritiis@gmail.com

1 Hyperparameters

We include hyperparameters used to generate the different trainings presented throughout the manuscript. Experiments were performed with the TorchMD-Net framework on two NVIDIA RTX 4090, except for Solvated Protein Fragments which was performed on four, using PyTorch Lightning's DDP multi-GPU training protocol.

Table S1: **Toy datasets training details and hyperparameters.**

Parameter	Value
`activation`	`silu`
`batch_size`	16
`cutoff_lower`	0.0
`cutoff_upper`	5.0
`derivative`	`True`
`early_stopping_patience`	100
`embedding_dimension`	128
`equivariance_invariance_group`	`O(3)`
`gradient_clipping`	40
`lr`	1e-3
`lr_factor`	0.5
`lr_min`	1e-7
`lr_patience`	15
`lr_warmup_steps`	0
`neg_dy_weight`	10.0
`num_layers`	2
`num_rbf`	32
`seed`	1
`train_size`	0.5
`val_size`	0.1
`y_weight`	1.0

Table S2: **SPICE PubChem training details and hyperparameters.**

Parameter	Value
activation	silu
batch_size	64
cutoff_lower	0.0
cutoff_upper	5.0
derivative	True
early_stopping_patience	50
embedding_dimension	128
equivariance_invariance_group	O(3)
gradient_clipping	100
lr	1e-3
lr_factor	0.5
lr_min	1e-7
lr_patience	5
lr_warmup_steps	0
neg_dy_weight	10.0
num_layers	2
num_rbf	32
seed	1
train_size	0.8
val_size	0.1
y_weight	1.0

Table S3: **Carbon chain, silver clusters, and sodium chloride clusters training details and hyperparameters.** In this case, a linear fit was performed beforehand on the training set to obtain element-wise reference energies, which were subtracted from total energy labels. For $Ag_3^{+/-}$, `y_weight` was 10.0.

Parameter	Value
activation	silu
batch_size	4
cutoff_lower	0.0
cutoff_upper	6.0
derivative	True
early_stopping_patience	300
embedding_dimension	128
equivariance_invariance_group	O(3)
gradient_clipping	100
lr	1e-3
lr_factor	0.5
lr_min	1e-7
lr_patience	15
lr_warmup_steps	100
neg_dy_weight	1.0
num_layers	2
num_rbf	32
seed	1
train_size	0.9
val_size	0.1
y_weight	1.0

Table S4: **Solvated protein fragments training details and hyperparameters.**

Parameter	Value
activation	silu
batch_size	32
cutoff_lower	0.0
cutoff_upper	5.0
derivative	True
early_stopping_patience	50
embedding_dimension	128
equivariance_invariance_group	O(3)
gradient_clipping	100
lr	1e-3
lr_factor	0.5
lr_min	1e-7
lr_patience	10
lr_warmup_steps	0
neg_dy_weight	10.0
num_layers	2
num_rbf	32
seed	1
train_size	2560000
val_size	100000
y_weight	1.0

Table S5: **QMspin training details and hyperparameters.** In this case, a linear fit was performed beforehand on the training set to obtain element-wise reference energies, which were subtracted from total energy labels.

Parameter	Value
activation	silu
batch_size	16
cutoff_lower	0.0
cutoff_upper	5.0
derivative	False
early_stopping_patience	100
embedding_dimension	128
equivariance_invariance_group	O(3)
gradient_clipping	40
lr	1e-3
lr_factor	0.5
lr_min	1e-7
lr_patience	30
lr_warmup_steps	0
neg_dy_weight	0.0
num_layers	2
num_rbf	32
seed	1
train_size	20000
val_size	1000
y_weight	1.0
